# Supplementary material for: Barriers and practices in pain management for intubated patients: A study of critical care nurses in Southern West Bank hospitals
Source: PLoS One. 2025 Mar 25;20(3):e0320430. doi: 10.1371/journal.pone.0320430 (PMC11936293; doi:10.1371/journal.pone.0320430)
Supplement: S1 Questionnaire — (DOCX) [file pone.0320430.s001.docx]

**Study Questionnaire**

**Part I: Socio-demographic Factors**

Please answer the following questions and circle to the closest answer that matches your opinion.

1. Gender
   1. Male
   2. Female

1. Marital status
   1. Single
   2. Married
   3. Divorced
   4. Widow/er/ed

1. Years of practice in Intensive Care Units
   1. 6 months ≤ 1 year
   2. 1 ≤ 5 Years
   3. 5 ≤ 10 Years
   4. ≥ 10 Years
2. Age
   1. 23 - 28 years
   2. 28 - 33 Years
   3. 33 - 38 Years
   4. ˃38 Years

1. Please, state your level of academic education
   1. Diploma Degree
   2. Bachelor degree
   3. Higher than a bachelor's degree

1. Where do you work? (Hospital)

------------------------------------

1. Did you have previous pain education in the last 2 years?
   1. Yes
   2. No

**Part II: Knowledge and Attitudes Survey Regarding Pain**

**True/False – Circle the correct answer, please.** (Correct answers are in **bold** and ***italics***).

| T | ***F*** | 1. | Vital signs are always reliable indicators of the intensity of a patient‘s pain. |
| --- | --- | --- | --- |
| T | ***F*** | 2. | Because their nervous system is underdeveloped, children under two years of age have decreased pain sensitivity and limited memory of painful experiences. |
| T | ***F*** | 3. | Patients who can be distracted from pain usually do not have severe pain. |
| ***T*** | F | 4. | Patients may sleep in spite of severe pain. |
| T | ***F*** | 5. | Aspirin and other nonsteroidal anti-inflammatory agents are NOT effective analgesics for painful bone metastases. |
| ***T*** | F | 6. | Respiratory depression rarely occurs in patients who have been receiving stable doses of opioids over a period of months. |
| ***T*** | F | 7. | Combining analgesics that work by different mechanisms (e.g., combining an NSAID with an opioid) may result in better pain control with fewer side effects than using a single analgesic agent. |
| T | ***F*** | 8. | The usual duration of analgesia of 1-2 mg morphine IV is 4-5 hours |
| T | ***F*** | 9. | Opioids should not be used in patients with a history of substance abuse. |
| T | ***F*** | 10. | Elderly patients cannot tolerate opioids for pain relief. |
| T | ***F*** | 11. | Patients should be encouraged to endure as much pain as possible before using an opioid. |
| T | ***F*** | 12. | Children less than 11 years old cannot reliably report pain so clinicians should rely solely on the parent‘s assessment of the child‘s pain intensity. |
| ***T*** | F | 13. | Patients‘spiritual beliefs may lead them to think pain and suffering are necessary. |
| ***T*** | F | 14. | After an initial dose of opioid analgesic is given, subsequent doses should be adjusted in accordance with the individual patient‘s response. |
| T | ***F*** | 15. | Giving patients sterile water by injection (placebo) is a useful test to determine if the pain is real. |
| ***T*** | F | 16. | Vicodin (hydrocodone 5 mg + acetaminophen 300 mg) PO is approximately equal to 5-10 mg of morphine PO. |
| T | ***F*** | 17. | If the source of the patient‘s pain is unknown, opioids should not be used during the pain evaluation period, as this could mask the ability to correctly diagnose the cause of pain. |
| T | ***F*** | 18. | Anticonvulsant drugs such as gabapentin (Neurontin) produce optimal pain relief after a single dose. |
| ***T*** | F | 19. | Benzodiazepines are not effective pain relievers and are rarely recommended as part of an analgesic regiment. |
| ***T*** | F | 20. | Narcotic/opioid addiction is defined as a chronic neurobiological disease, characterized by behaviors that include one or more of the following: impaired control over drug use, compulsive use, continued use despite harm, and craving. |
| ***T*** | F | 21. | The term ‗equianalgesia‘means approximately equal analgesia and is used when referring to the doses of various analgesics that provide approximately the same amount of pain relief. |
| ***T*** | F | 22. | Sedation assessment is recommended during opioid pain management because excessive sedation precedes opioid-induced respiratory depression. |

**Multiple Choice – Place a check by the correct answer** (Correct answers are in **bold** and *italics*).

1. The recommended route of administration of opioid analgesics for patients with persistent cancer-related pain is
   1. Intravenous
   2. Intramuscular
   3. Subcutaneous
   4. ***Oral***
   5. Rectal

1. The recommended route administration of opioid analgesics for patients with brief, severe pain of sudden onset such as trauma or postoperative pain is

***a. Intravenous***

- 1. Intramuscular
  2. Subcutaneous
  3. Oral
  4. Rectal

1. Which of the following analgesic medications is considered the drug of choice for the treatment of prolonged moderate to severe pain for cancer patients?

a. Codeine

- 1. ***Morphine***
  2. Meperidine
  3. Tramadol

1. A 30 mg dose of oral morphine is approximately equivalent to:
   1. Morphine 5 mg IV
   2. ***Morphine 10 mg IV***
   3. Morphine 30 mg IV
   4. Morphine 60 mg IV

1. Analgesics for postoperative pain should initially be given.
   1. ***Around the clock on a fixed schedule***
   2. Only when the patient asks for the medication
   3. Only when the nurse determines that the patient has moderate or greater discomfort

1. A patient with persistent cancer pain has been receiving daily opioid analgesics for 2 months. Yesterday the patient was receiving morphine 200 mg/hour intravenously. Today he has been receiving 250 mg/hour intravenously. The likelihood of the patient developing clinically significant respiratory depression in the absence of new comorbidity is

***a. Less than 1%***

- 1. 1-10%
  2. 11-20%
  3. 21-40%
  4. > 41%

1. The most likely reason a patient with pain would request increased doses of pain medication is
   1. ***The patient is experiencing increased pain.***
   2. The patient is experiencing increased anxiety or depression.
   3. The patient is requesting more staff attention.
   4. The patient‘s requests are related to addiction.

1. Which of the following is useful for the treatment of cancer pain?
   1. Ibuprofen (Motrin)
   2. Hydromorphone (Dilaudid)
   3. Gabapentin (Neurontin)
   4. ***All of the above***

1. The most accurate judge of the intensity of the patient‘s pain is
   1. The treating physician
   2. The patient‘s primary nurse
   3. ***The patient***
   4. The pharmacist
   5. The patient‘s spouse or family

1. Which of the following describes the best approach for cultural considerations in caring for patients in pain:
   1. There are no longer cultural influences in Palestine due to the diversity of the population.
   2. ***Patients should be individually assessed to determine cultural influences.***
   3. Cultural influences can be determined by an individual ‘s socioeconomic status (e.g., blue-collar workers report more pain than white-collar workers do).

1. How likely is it that patients who develop pain already have an alcohol and/or drug abuse problem?

1. < 1%
2. ***5 – 15%***
3. 25 - 50%
4. 75- 100%

1. The time to peak effect for morphine given IV is
   1. ***15 min.***
   2. 45 min.
   3. 1 hour
   4. 2 hours
2. The time-to-peak effect of morphine given orally is
   1. 5 min.
   2. 30 min.
   3. ***1 – 2 hours***
   4. 3 hours

1. Following the abrupt discontinuation of an opioid, physical dependence is manifested by the following:
   1. ***Sweating, yawning, diarrhea, and agitation with patients when the opioid is abruptly discontinued.***
   2. Impaired control over drug use, compulsive use, and craving.
   3. A need for higher doses to achieve the same effect.
   4. A and b
2. Which statement is true regarding opioid-induced respiratory depression?
   1. More common several nights after surgery due to the accumulation of opioids.
   2. ***Obstructive sleep apnea is an important risk factor.***
   3. Occurs more frequently in those already on higher doses of opioids before surgery.
   4. Can be easily assessed using intermittent pulse oximetry.

**Case Studies**

Two patient case studies (A and B) are presented. For each patient, you are asked to make decisions about pain and medication.

**Directions:** Please select one answer for each question.

**Patient A**: Ahmad is 25 years old, and this is his first day following abdominal surgery. As you enter his room, he smiles at you and continues talking and joking with his visitor. Your assessment reveals the following information: BP = 120/80; HR = 80; R = 18; on a scale of 0 to 10 (0 = no pain/discomfort, 10 = worst pain/discomfort).

1. **A.** On the patient‘s record, you must mark his pain on the scale below. Circle the number that represents your assessment of Ahmad‘s pain.

0 1 2 3 4 5 6 7  ***8*** 9 10

---------------------------------------------------------------------------------------------

No pain/discomfort Worst

Pain/discomfort

1. **B.** Your assessment, above, is made two hours after Ahmad received morphine 2 mg IV. Half-hourly pain ratings following the injection ranged from 6 to 8 and he had no clinically significant respiratory depression, sedation, or other untoward side effects. He has identified 2/10 as an acceptable level of pain relief. His physician ‘s order for analgesia is ―morphine IV 1-3 mg q1h PRN pain relief.‖ Check the action you will take at this time.
   1. Administer no morphine at this time.
   2. Administer morphine 1 mg IV now.
   3. Administer morphine 2 mg IV now.
   4. ***Administer morphine 3 mg IV now.***

**Patient B**: Mohammad is 25 years old and this is his first day following abdominal surgery. As you enter his room, he is lying quietly in bed and grimaces as he turns in bed. Your assessment reveals the following information:

BP = 120/80; HR = 80; R = 18; on a scale of 0 to 10 (0 = no pain/discomfort, 10 = worst pain/discomfort).

1. **A.** On the patient‘s record, you must mark his pain on the scale below. Circle the number that represents your assessment of Robert‘s pain:

0 1 2 3 4 5 6 7  ***8*** 9 10

---------------------------------------------------------------------------------------------

No pain/discomfort Worst Pain/discomfort

1. **B.** Your assessment, above, is made two hours after Mohammad received morphine 2 mg IV. Half-hourly pain ratings following the injection ranged from 6 to 8 and he had no clinically significant respiratory depression, sedation, or other untoward side effects. He has identified 2/10 as an acceptable level of pain relief. His physician‘s order for analgesia is ―morphine IV 1-3 mg q1h PRN pain relief.‖ Check the action you will take at this time:
   1. Administer no morphine at this time.
   2. Administer morphine 1 mg IV now.
   3. Administer morphine 2 mg IV now.
   4. **Administer morphine 3 mg IV now.**

1. From your perception, how can you improve pain management in ICUs?

**………………………………………………………………………………………………….**

**………………………………………………………………………………………………….**

**Part IV: Mean of Nurses’ Perceived Barriers to Assessment and Management of Pain Barriers**

| **No:** | **Statement** | | **Agree** | **Disagree** | **Do not know** |
| --- | --- | --- | --- | --- | --- |
| 1. | **Patients**  **Related barriers** | **Patients‘ difficulty with completing pain scales (eg, 010)** |  |  |  |
| 2. |  | **Consumers not demanding results** |  |  |  |
| 3. |  | **Patients‘ reluctance to take pain medication for fear of addiction** |  |  |  |
| 4. |  | **Caregiver‘s indifference** |  |  |  |
| 5. |  | **Patients‘ reluctance to take pain medications because of side effects (e.g. constipation, how it makes them feel, etc.)** |  |  |  |
| 6. |  | **Patients reporting their pain to the doctor, but not to the nurse** |  |  |  |
| 7. |  | **Patient‘s reluctance to take opioids** |  |  |  |
| 8. |  | **Patient‘s reluctance to report pain** |  |  |  |
| 9. |  | **Patients not wanting to bother the nurses** |  |  |  |
| 10. | **Nurses Related**  **Barriers** | **Inadequate time for health teaching with patients (eg, as needed drug order, alternatives, addiction, etc.)** |  |  |  |
| 11. |  | **Inadequate time to deliver non-pharmacologic pain relief measures** |  |  |  |
| 12. |  | **Inadequate staff knowledge of pain management** |  |  |  |
| 13. |  | **Nursing staff reluctance to administer opiates** |  |  |  |
| 14. |  | **Fear of pain medications because of side effects** |  |  |  |
| 15. |  | **Inadequate assessment of pain** |  |  |  |
| 16. |  | **Nurses indifference** |  |  |  |
| 17. | **Physician Related barriers** | **Inadequate assessment of pain and pain relief** |  |  |  |
| 18. |  | **Doctor‘s indifference** |  |  |  |
| 19. |  | **Physicians‘ reluctance to prescribe opiates because of the side effects** |  |  |  |
| 20. |  | **Inadequate knowledge of pain management** |  |  |  |
| 21. |  | **Physicians‘ fear of addiction of medicine** |  |  |  |
| 22. |  | **Physicians‘ reluctance to prescribe adequate pain relief for fear of overmedicating** |  |  |  |
| 23. |  | **Physicians‘ lack of trust in the nursing assessment of pain** |  |  |  |
| 24. | **System Related**  **Barriers** | **Lack of psychosocial support services** |  |  |  |
| 25. |  | **Patient-to-nurse ratio** |  |  |  |
| 26. |  | **Lack of social workers who are experienced in hospital settings** |  |  |  |
| 27. |  | **Lack of guidelines for pain management** |  |  |  |
| 28. |  | **Lack of access to professionals who practice specialized pain treatment methods** |  |  |  |
| 29. |  | **Difficulty contacting or communicating with**  **physicians to discuss treatment of pain in patients** |  |  |  |
| 30. |  | **Not having a documented pain treatment plan for each patient** |  |  |  |
| 31. |  | **Lack of alternatives non-pharmacologic therapy for pain Management (cold, hot, acupuncture) Narcotic prescription regulation** |  |  |  |
| 32. |  | **Inconsistent practices around giving as needed medications for patient** |  |  |  |
| 33. |  | **Lack of medicine in markets** |  |  |  |
| 34. |  | **Lack of equipment or skill in using equipment** |  |  |  |
