# Supplementary material for: Barriers and practices in pain management for intubated patients: A study of critical care nurses in Southern West Bank hospitals
Source: PLoS One. 2025 Mar 25;20(3):e0320430. doi: 10.1371/journal.pone.0320430 (PMC11936293; doi:10.1371/journal.pone.0320430)
Supplement: Attachment — Submitted filename: Response to Reviewers.pdf [file pone.0320430.s003.pdf]

Response to Reviewers

Items to Address	Authors' Response
**Title and Abstract**	
**Clarity in Title:** The title is too descriptive; it needs to be shortened to make it more readable, such as: *"Barriers and Practices in Pain Management for Intubated Patients: A Study of Critical Care Nurses in Southern West Bank Hospitals."*	**Authors' Response:** The title has been revised for clarity and conciseness. The new title, **"Barriers and Practices in Pain Management for Intubated Patients: A Study of Critical Care Nurses in Southern West Bank Hospitals,"** accurately reflects the study while improving readability.
**Abstract Improvements:** 1) Include a particular statistic or outcome in the results section to make it more effective. 2) Briefly mention the implications for patient care and health policies.	**Authors' Response:** We have modified the abstract by incorporating key statistical results, including the percentage of nurses with inadequate knowledge (96.5%) and key systemic barriers (mean score of 2.41/3). Additionally, we have added a brief mention of the study's implications for patient care and health policies.
**Introduction**	
**Contextual Background:** 1) Establish a broader importance of the study by connecting it to global trends in pain management. 2) Clearly explain why the focus on the Southern West Bank is unique and important.	**Authors' Response:** The introduction has been revised to explicitly relate the study to global pain management challenges, referencing WHO and IASP guidelines. Additionally, we clarified why pain management in intubated patients in the Southern West Bank presents unique challenges due to healthcare constraints and inconsistent protocols.
**Research Gap:** The researcher needs to emphasize gaps in previous studies to justify the research.	**Authors' Response:** The research gap has been clearly articulated, emphasizing the lack of studies in the Southern West Bank that assess systemic, physician-related, and nurse-related barriers to pain management. Justification for the study's relevance has been strengthened.
**Methods**	
Details about hospital settings and patient profiles should be included to enhance generalizability.	**Authors' Response:** The Methods section has been expanded to describe the participating hospitals, their ICU capacities, and the patient population they serve. These details improve the study's generalizability.
**Sampling Justification:** Provide a justification for using convenience sampling and discuss the biases it may introduce.	**Authors' Response:** We have explicitly acknowledged the potential selection bias due to convenience sampling and discussed its limitations in the *Methods* and *Limitations* sections. Additionally, we justified its use due to logistical constraints and access to ICU nurses during working shifts.
**Instrument Validation:** Explain how the	**Authors' Response:** The instrument validation

NKASRP and modified barriers scale were adapted for the Southern West Bank, considering cultural and linguistic aspects.	section has been expanded to describe how the NKASRP and the Perceived Barriers to Pain Management Scale were culturally and linguistically adapted for the Southern West Bank context. The translation and expert validation processes are now detailed.
**Discussion**	
**Critical Analysis:** 1) Provide a deeper comparison of the identified barriers with international pain management guidelines. 2) Explain potential reasons why nurses lack knowledge and face inadequate training.	**Authors' Response:** We have enhanced the discussion by comparing our findings with WHO and IASP guidelines, highlighting systemic gaps in training and protocol adherence. Additionally, we have explained how limited access to continuing education, workload pressures, and regulatory issues contribute to nurses' inadequate knowledge.
**Wider Implications:** Relate findings to global healthcare and their applicability outside the regional focus.	**Authors' Response:** The discussion now draws connections between the Southern West Bank's challenges and those faced in other middle- and low-income countries, emphasizing the global need for structured pain management training and interdisciplinary collaboration.
**Alternative Solutions:** Suggest more innovative and region-specific recommendations, such as telehealth for pain management training.	**Authors' Response:** We have added specific, region-focused recommendations, including telehealth-based pain management training, AI-driven pain assessment tools, and mobile-based pain assessment applications to support real-time clinical decision-making.
**Conclusion**	
**Conciseness:** The conclusion should be more concise while specifying actionable steps for practitioners and policymakers.	**Authors' Response:** The conclusion has been streamlined to focus on clear, actionable steps, emphasizing the need for structured training programs, standardized pain management protocols, and interdisciplinary collaboration.
**Future Research Directions:** Clearly state how future studies can address identified limitations, such as causal relationships or longitudinal outcomes.	**Authors' Response:** We have specified the need for longitudinal studies to assess the long-term impact of pain management interventions and interventional studies to explore causal relationships. These additions enhance the future research implications.
**References**	
**Formatting Consistency:** Ensure all references follow a consistent citation style (e.g., APA or Vancouver) and verify DOIs/links for accessibility.	**Authors' Response:** We have checked and standardized all references to adhere to Vancouver style, ensuring consistency. Additionally, DOIs and links have been verified for accessibility.
**Recent Literature:** Incorporate more recent studies (2023–2024) to strengthen the discussion on global pain management	**Authors' Response:** New references from 2023–2024 have been incorporated in the discussion to reflect recent advancements in pain management

trends.	training, AI-based assessment tools, and digital learning strategies.
**Editor's Additional Comments**	
**Title Revision:** Refine the title to be **more concise and engaging.**	**Authors' Response:** The title has been revised as suggested to improve clarity and engagement.
**Discussion Enhancement:** Compare findings more explicitly with international guidelines and discuss broader implications.	**Authors' Response:** The discussion now explicitly compares results with international standards (WHO, IASP) and highlights broader implications for global healthcare.
**Methodological Justification:** Provide more details on the methods section, answering the 5W (Who, What, Where, When, Why) questions.	**Authors' Response:** The Methods section has been expanded to address all 5W questions, ensuring a clear justification of study design and procedures.
**Selection Bias:** Either acknowledge its limitations or justify why the sample remains representative.	**Authors' Response:** Selection bias has been explicitly acknowledged in the limitations section, and justifications for sample representativeness have been provided.
**Response Rate Calculation:** Correct the response rate calculation based on the total number of critical care nurses in participating facilities.	**Authors' Response:** The response rate has been recalculated to reflect the total number of critical care nurses in the participating hospitals rather than only those who received questionnaires.
**Subgroup Analyses:** Clearly describe all subgroup analyses in the Methods section, including rationale and statistical methods used.	**Authors' Response:** The Methods section now explicitly details the rationale, statistical methods, and variables considered in subgroup analyses.
**Generalizability Consideration:** Discuss how selection bias limits generalizability and suggest how future studies could improve external validity.	**Authors' Response:** The limitations section now explicitly addresses selection bias and suggests future research directions, including randomized sampling and multicenter studies to enhance external validity.
