# Supplementary material for: Barriers and practices in pain management for intubated patients: A study of critical care nurses in Southern West Bank hospitals
Source: PLoS One. 2025 Mar 25;20(3):e0320430. doi: 10.1371/journal.pone.0320430 (PMC11936293; doi:10.1371/journal.pone.0320430)
Supplement: Attachment — Submitted filename: Article review.docx [file pone.0320430.s002.docx]

Thank you for the opportunity to review this manuscript entitled: Knowledge, Attitudes, Practice, and Perceived Barriers of Critical Care Nurses Regarding Pain Management for Intubated Patients in xxx Hospitals"

Generally, the manuscript is literally sound with minor errors in punctuation and grammar. The manuscript addresses an important topic. I hope the suggestions will be considered to strengthen the quality of the manuscript.

**Title and Abstract**

1.	Clarity in Title: The title is too descriptive; it needs to be shortened to make it more readable, such as: "Barriers and Practices in Pain Management for Intubated Patients: A Study of Critical Care Nurses in Southern West Bank Hospitals."

2.	Abstract Improvements:

o	An included particular statistic or an outcome in the results section of the abstract will make it more effective.

o include a brief mention of implications that can be made from the findings, especially for patient care and health policies.

**Introduction**

1.

Contextual Background:

o The findings of the study were set against the backdrop of global trends in pain management in intubated patients to establish a broader importance of the study.

o Clearly explain why the focus is particularly unique or important in the Southern West Bank.

2.

Research gap: The researcher needs to emphasize some gaps regarding previous studies and, therefore explains the justification of research.

**Methods**

1

Details about selected setting of hospitals and patient profile in those can help in getting better generalisation.

2. Sampling:

Justification for convenient sampling and commenting on what kind of biases it will bring into samples

3. Instrument Validation:

o	Shortly discuss how the NKASRP and modified barriers scale were adapted for the specific context of Southern West Bank hospitals, including cultural and linguistic considerations.

**Discussion**

1.

Critical Analysis:

o	Discuss deeper the comparison of the identified systemic and attitudinal barriers in relation to international standards or guidelines on pain management.

o	Explain potential reasons why nurses have a lack of knowledge and face inadequate training opportunities in the region.

2.

Wider Implications:

o Relate findings to global healthcare and their applicability outside of the regional focus.

3.

Alternative Solutions:

o Put forward more innovative or region-specific recommendations for overcoming the identified barriers, such as the use of telehealth for pain management training.

**Conclusion**

1.

Briefness: It would be better if the conclusion was concise, yet specified the actionable steps for practitioners and policymakers.

2.

Future Research Directions: Clearly state how future studies may investigate the limitations identified, for instance, in investigating causal relationships or longitudinal outcomes.

**References**

1.	Formatting Consistency: Ensure all references adhere to the same citation style (e.g., APA or Vancouver) and verify DOIs or links for accessibility.

2.	Recent Literature: Incorporate more recent studies from 2023–2024 to strengthen the study's relevance, particularly in sections discussing global pain management trends.
